# Phytochemical and Antimicrobial Studies of Methyl Angolensate and Luteolin-7-*O*-glucoside Isolated from Callus Cultures of *Soymida febrifuga*

**Published:** 2007-12

**Authors:** Kishore K. Chiruvella, Arifullah Mohammed, Gayathri Dampuri, Rama Gopal Ghanta, Sathees C. Raghavan

**Affiliations:** 1 *Division of Plant Tissue Culture, Department of Botany, Sri Venkateswara University, Tirupati-517502, Andhra Pradesh, India;*; 2 *Department of Biochemistry, Indian Institute of Science, Bangalore, India*

**Keywords:** *Soymida febrifuga*, antibacterial, antifungal, silica gel column chromatography, callus, methyl angolensate, luteolin-7-*O*-glucoside, Meliaceae

## Abstract

*Soymida febrifuga* (Roxb.) A. Juss. is an indigenous lofty deciduous medicinal tree, monotypic genus endemic to India. Hexane, ethyl acetate and methanol extracts of *Soymida febrifuga* root callus were tested for their phytochemical constituents and antimicrobial activity. Among them, ethyl acetate extract was found to be most effective, which on subjection to silica gel column chromatography led to the separation and isolation of methyl angolensate and luteolin-7-*O*-glucoside. Structures were determined by Nuclear Magnetic Resonance and Liquid Chromatographic Mass Spectroscopic methods. Further studies indicated that methyl angolensate and luteolin-7-*O*-glucoside had an anti-bacterial effect against *Bacillus subtilis* and *Salmonella typhimurium,* respectively. In addition to that methyl angolensate had an anti-fungal activity against *Aspergillus niger* while luteolin-7-*O*-glucoside inhibited *Alternaria alternata.*

## INTRODUCTION

Wild plant species are excellent sources of a wide range of biochemicals. Currently many of these compounds are isolated by solvent extraction from naturally grown whole plants. The continued destruction of plants due to environmental and geopolitical instability poseshas posed a major threat and is increasingly difficult to acquire plant-derived compounds. Clearly, the development of alternative methods to whole plant extraction for the production of compounds of medicinal value is an issue of considerable socio-economic importance. This has prompted scientists to consider alternative methods for production of secondary plant metabolites. *In vitro* cultures have long been recognised as a means of avoiding many of these problems. Theoretically by growing undifferentiated tissues *in vitro* large amounts of biosynthetically active tissue could be generated under conditions in which both seasonality and time specificity of production could be circumvented.

*Soymida febrifuga* (Roxb.) A. Juss. (Meliaceae) is an indigenous lofty deciduous medicinal tree endemic to India (1). The decoction of the bark contains a resinous bitter principle well adapted for gargles, vaginal infections, enemata, rheumatic swellings, and stomach pain. The bark is said to be used as an anti-cancer remedy (2), for blood coagulation, wounds, dental diseases, uterine bleeding and haemorrhage (3) and acrid, refrigerant, antihelminthic, aphrodisiac, laxative; good for sore throat; removes “vata”; cures ‘tridosha’ fevers, cough, asthma (4) and is anti-inflammatory (5) in action. To our knowledge, studies aimed at investigating the secondary metabolite production of *S. febrifuga* tissue cultures have not yet been carried out. In the present study, we examine the phytochemical activity of metabolites in callus extracts, isolate phytochemical constituents by column chromatography, characterize isolated compounds by spectral studies and analyse antimicrobial activity of the extracts and isolated compounds.

## MATERIALS AND METHODS

### *In vitro* Cultures of *S. febrifuga*

Seed explants were disinfected with 0.05 % (v/v) Tween 80 solutoin for 10 min with agitation and rinsed three times with sterile distilled water. Roots were excised from 12-15 day aseptic seedling and cultured on MS medium supplemented with 30 g/L^-1^ sucrose and different combinations of 2, 4-Dichlorophenoxyacetic acid alone or in combination with benzyl amino purine/2-isopentenyl adenine. The pH value was adjusted to 5.8 prior to autoclaving (121°C, 15 min) and the medium was solidified with 8 g/L^-1^ agar (Sigma). The callus cultures were grown at 25 ± 2°C under white fluorescent light with 16h photoperiod. Subcultures of calli to fresh medium with the same composition were made at monthly intervals during a period of 3 months.

### Soxhlet Extraction of Callus and Phytochemical Screening

The procured calli were first dried at room temperature for a few days and the material was then crushed to a fine powder. 600 g of dried and powdered callus was placed in a 500 ml soxhlet glass thimble and extraction was carried out using hexane, ethyl acetate and methanol. Extracts obtained with each solvent were collected separately. All crude fractions were tested for the detection of secondary metabolites such as flavonoids, phenols, quinones, terpenoids, tannins (6-10).

### Isolation by Silica Gel Column Chromatography

The dry crude ethyl acetate extract was dissolved in a minimum amount of ethyl acetate and adsorbed on silica gel (100 to 200 mesh size) to form a slurry. The slurry was dried under reduced pressure in rota-vapor. A column was prepared using silica gel (100 to 200 mesh size) ten to twenty times the weight of dry crude extract in ethyl acetate by dry packing method. The dry slurry was loaded onto the prepared column (100 cm × 3.5 cm i.d.) and the column was eluted initially in hexane followed by hexane: ethyl acetate along a linear gradient and finally exhausted using 100% ethyl acetate. Fraction of 75 ml were collected, concentrated and left for crystallization. Purification of isolated compound was carried out by washing and recrystallization technique. Fractions were monitored on silica gel (G) TLC. Visualization of spots on TLC plates was carried out either under UV light or by exposing TLC plates to iodine vapours. Similar fractions were combined and the results recorded.

### Antibacterial and Antifungal Assays

*In vitro* antibacterial and antifungal assays were carried out by adopting the agar well diffusion method (11) for the crude extracts and standard disc diffusion technique (12) for the isolated compounds on Mueller-Hinton agar (MHA) for bacteria and Sabouraud Dextrose Agar (SDA) for the fungus. Microorganism innocula were prepared separately from 12 h broth cultures. Except otherwise stated, all culture media and distilled water were sterilized at 121°C for 15 min in an autoclave. These inocula were diluted with sterilized distilled water to obtain a density corresponding approximately to 0.5 of Mc Farland standard turbidity scale (10^8^ colony forming unit “CFU” per ml for the bacteria and 10^3^ spores per ml for fungi). Sterile discs of 6mm in diameter were made from Fisher filter paper P5 (Catalog No.09-801C). The discs were impregnated with test compounds methyl angolensate (10 mg/ml) and luteolin-7-*O*-glucoside (10 mg/ml). The reference drugs were ciprofloxacin (10 g/ml) and nystatin (500 g/ml). After moistening the discs, they were immediately transferred to the inoculated solid media. Each test concentration had three replications. The plates were incubated at 37°C for 24 and 48 h for the bacteria and fungus, respectively. The results were recorded as the mean diameter of the zones of growth inhibition surrounding the discs. Bacterial and fungal strains used were *Klebsiella pneumoniae, Salmonella typhimurium, Proteus vulgaris, Pseudomonas aeruginosa, Bacillus subtilis, Escherichia coli, Staphylococcus aureus, Aspergillus fumigatus, Aspergillus niger*, and *Alternaria alternate*. These were obtained from the stock culture of Pathology Laboratory, Sri Venkateswara Institute of Medical Sciences, Tirupati and Microbiology Laboratory, S.V. University, Tirupati, Andhra Pradesh, India.

## RESULTS AND DISCUSSION

### Preliminary Phytochemical Screening

Powdered callus was subjected to soxhletion with hexane, ethyl acetate and methanol to obtain their respective soluble fractions. These were qualitatively screened for the occurrence of various secondary metabolites such as flavonoids, terpenoids, alkaloids, glycosides, sterols, saponins, phenols, lignin and tannins. All the extracts were negative for the presence of alkaloids when detected by Mayers, Dragendorffs and Wagners tests (Table 1). Flavonoids were detected using Shinoda’s, zinc HCl reduction and lead acetate tests. All three extracts showed the presence of tannins and quinones whereas saponins were found to be absent. Various tests showed that ethyl acetate and methanol extracts were chemically diversified (Table 1). Preliminary phytochemical studies are of importance because the crude extracts possess varied composition of secondary metabolites (13, 14).

**Table 1:** Preliminary screening of secondary metabolites from callus of *S. febrifuga*

Tests for Secondary metabolites		Hexane	Ethyl acetate	Methanol

**Alkaloids**	Mayers test	-	-	-
	Wagners test	-	-	-
	Dragendorff’s test	-	-	-
**Flavonoids**	FeCl_3_ test	+	+	+
	Shinoda’s test	+	+	+
	Zinc HCl reduction test	+	+	+
	Lead acetate	+	+	+
**Phenols**	Phenol test	-	+	+
	Ellagic test	-	+	+
**Glycosides**	Kellar Kilani test	-	+	-
**Lignin**	Labat test	-	-	-
	Lignin test	-	-	-
**Tannins**	Gelatin test	+	+	+
	FeCl_3_ test	+	+	+
	Lead acetate	-	-	-
**Terpenoids**		-	+	----
**Quinones**		----	+	----
**Saponins**		-	+	-

+, indicates presence; -, absence.

The biological activity of the callus crude extracts (hexane, ethyl acetate and methanolic extracts) was determined by antibacterial and antifungal assay using agar-well diffusion method. Among the different callus extracts of *S. febrifuga*, the ethyl acetate and methanol extract were found to exhibit growth inhibition of seven and six selected organisms respectively, whereas the hexane extract exhibited inhibition of three of the seven organisms (data not shown). The ethyl acetate extract was subjected to silica gel column chromatography for the isolation of the phytochemical constituents (Table 2). It is well known that callus cultures are able to produce secondary metabolites, sometimes even in quantities that allow economically feasible production than the cell cultures (15). The products isolated from the callus cultures are produced in high levels (16) and their yields largely depend on the culture conditions (17). The biochemical mechanisms of induction and stimulation of secondary metabolites synthesis by growth regulators are not clear. They may act by repressing, stimulating or inducting a common precursor or transforming an intermediate compound involved in the biosynthetic pathway, resulting in differentiated metabolite production patterns with regard to the original explants (18).

**Table 2:** Silica gel column chromatography of ethyl acetate callus extract of *S. febrifuga*

Eluent (Hexane:EtOAc)	Fractions	Remarks

100:0	1-8	Fatty oil
90:10	9-20	Fatty oil
80:20	21-30	Fatty oil
70:30	31-35	**Fraction-A**
60:40	36-41	Brownish gum
50:50	42-50	Brownish gum
40:60	51-60	Brownish gum
30:70	61-70	Intractable gum
20:80	71-80	Intractable gum
10:90	80-90	Intractable gum
0:100	91-100	**Fraction-B**

### Examination of Ethyl Acetate Extract by Silica Gel Column Chromatography

Ethyl acetate extract upon concentration yielded a brown viscous residue (40 g). This residue was subjected to column chromatography over silica gel using hexane: ethyl acetate mixtures in increasing polarity. Fraction A and fraction B were collected using hexane:ethyl acetate (7:3) and ethyl acetate respectively (Table 2). Fractions (1-30 and 36-90) on concentration yielded oil and gums, which were not examined further.

The fraction A is a mixture of compounds which was obtained from silica gel TLC plates and was rechromatographed over silica gel (100-200 mesh) column using hexane: ethyl acetate mixtures as eluents. Fractions 21-30 obtained with hexane: ethyl acetate (8:2) upon concentration gave colourless crystalline needles (1500 mg). The other fractions (1-20 and 31-60) did not give any crystalline compounds, hence they were not investigated. The fraction B obtained from silica gel TLC plates was rechromatographed over silica gel (100-200 mesh) column using ethyl acetate: methanol mixtures as eluents. Fractions 11-25 of ethyl acetate: methanol (9:1) upon concentration gave a yellow amorphous powder (130 mg). Fractions 1-10 and 26-60 on evaporation did not yield any crystalline principle.

### Structure Elucidation of Isolated Compounds

Colourless needles purified from fraction A were analyzed by LC-MS which showed molecular ion peak at m/z 470. This was further supported by ^13^C NMR spectrum, which showed signals for 34 carbons present in the molecule. The IR spectrum showed absorption bands at 1736, 1570 and 850 cm^-1^ indicating the presence of lactone and β-substituted furan ring, respectively.

IR (KBr) ν_max_ : 3421, 2965, 1736 (lactone), 1510 (furan), 1461, 1388, 1360, 1274, 1242, 1187,1136, 1028, 903, 850 cm^-1^.

**Figure 1:**
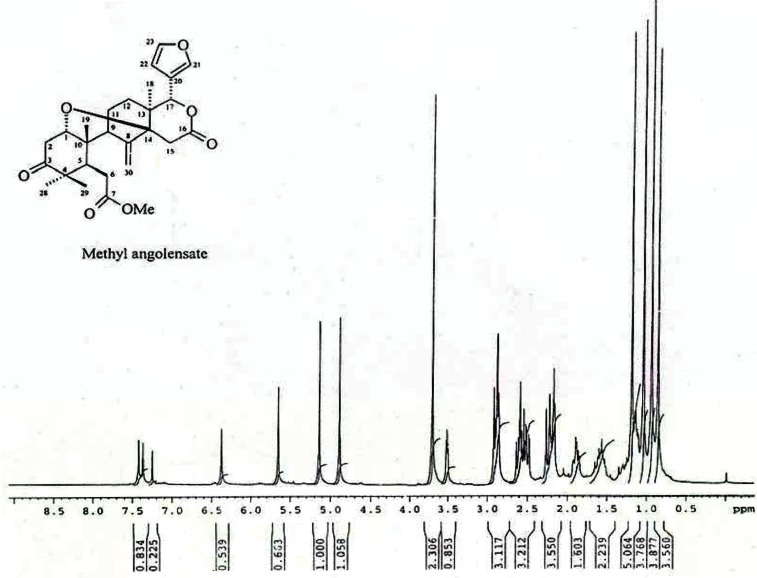
^1^H NMR spectra of methyl angolensate (400 MHz, CDCL_3_) showing a single at δ 3.70 corresponds to three protons of methoxyl group. It also shows three low field protons at δ 5.65, 4.88, 5.13 due to H-17, and methylene protons (CH_2_-30) and four tertiary methyl group at δ 1.18 (Me-29), 1.03 (Me-28), 0.93 (Me-19) and 0.85 (Me-18). Band at δ 3.51 indicates the presence of a cyclic ether [(0.85 and 0.93- angular methyls), (1.18 and 1.03- gem-dimethyls)]. The protons of the furan ring appear as singlet at δ 7.42, 7.37 and 6.37 respectively.

^1^H NMR (400 MHz, CDCl_3_) (Fig. 1): δ 7.42 (1H, dd, *J* = 1.5, 0.8 Hz, H-21), 7.37 (1H, m, H-23), 6.37 (1H, dd, *J* = 1.5, 0.8 Hz, H-22), 5.65 (1H, s, H-17), 5.13 (1 H, s, CH_2_-30), 3.70 (3H, s, OMe), 3.51 (1H, dd, *J* = 6.5, 4.0 H_2_, H-1), 2.91 (2H, d, *J* = 18 Hz, CH_2_-15), 2.89 (2H,dd, *J* = 14.5, 6.0 Hz, CH_2_- 2), 2.87 (1H, d, *J* = 10.5 Hz, H-5), 2.60 (2H, dd, *J* = 16.5, 10.5 Hz, CH_2_-6), 2.22 (2H, m, CH_2_- 11), 2.17 (1H, dd, *J* = 5.0, 1.5 Hz, H-9), 1.89 (2H, d, *J* = 14.0, 5.0 Hz, CH_2_- 12), 1.18 (3H, s, CH_3_- 29), 1.03 (3H, s, CH_3_- 19), 0.85 (3H, s, CH_3_- 18).

**Figure 2:**
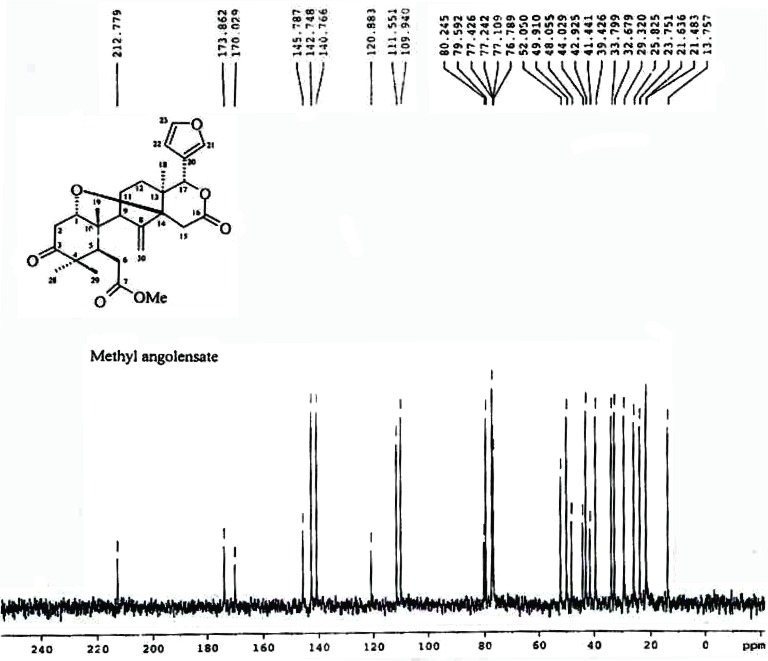
^13^C NMR spectra of methyl angolensate (400 MHz, CDCL_3_) showing signals for all the 34 carbons present in the molecule. Protons attached to C-1 carbon appearing as double doublet signal at δ 3.51 due to coupling with the neighbouring hydrogen atoms at C-2. The presence of a double bond shows that methyl angolensate has two carbocyclic rings.

^13^C NMR (400 MHz, CDCl_3_) (Fig. 2): δ 212.7 (C-3), 173.8 (C-7), 170.0 (C-16), 145.7 (C-8), 142.7 (C-23), 140.7 (C-21), 120.8 (C-20), 111.5 (C-30), 109.9 (C-22), 80.2 (C-14), 79.5 (C-17), 76.7 (C-1), 52.0 (COOMe), 49.9 (C-9), 48.0 (C-4), 44.0 (C-10), 42.9 (C-5), 41.4 (C-13), 39.4 (C-2), 33.7 (C-15), 32.6 (C-6), 29.3 (C-12), 25.8 (C-28), 23.7 (C-11), 21.6 (C-19), 21.4 (C-29), 13.7 (C-18). LC-MS (M + H)^+^ 470 (M + Na)^+^ 493

**Figure 3:**
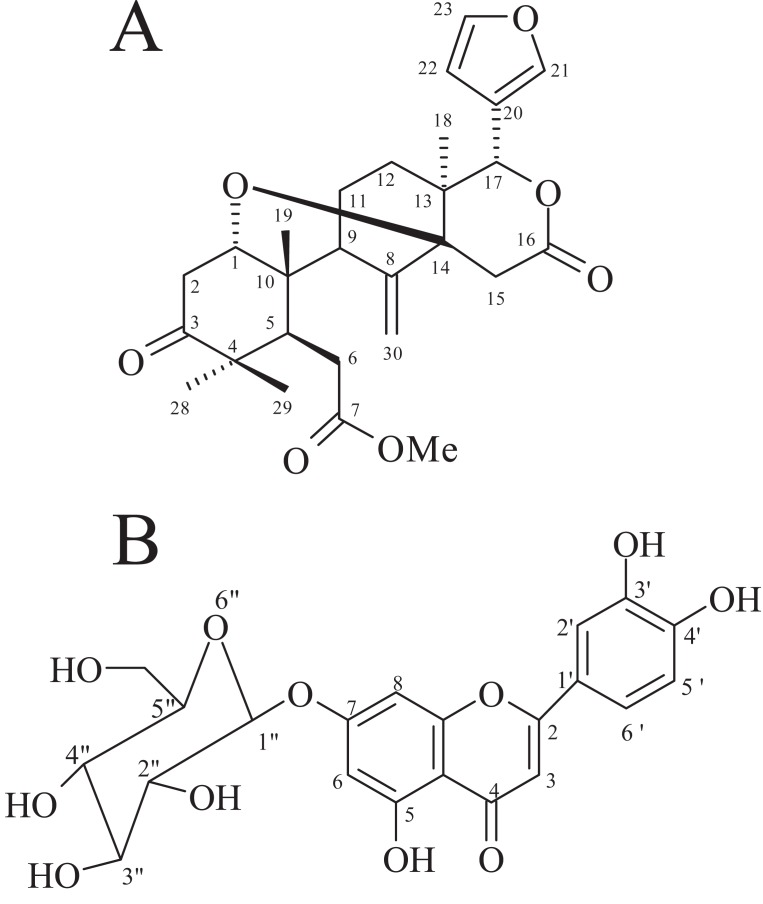
(A), Structure of Methyl Angolensate; (B), Structure of Luteolin-7-*O*-glucoside.

The ^1^H NMR spectrum showed a singlet at δ 3.70 corresponding to the three protons of methoxyl group. The ^1^H NMR spectrum showed bands due to four tertiary methyl groups at δ 1.18 (Me-29), 1.03 (Me-28), 0.93 (Me-19) and 0.85 (Me-18). It also had three low field protons at δ 5.65, 4.88 and 5.13 due to H-17, and methylene protons (CH_2_-30), respectively (Fig 1). The ^1^H NMR spectrum had a band at δ 3.51 due to one proton, indicating the presence of cyclic ether ((0.85 and 0.93- angular methyls), (1.18 and 1.03- gem-dimethyls)). The protons of the furan ring appear as singlet at δ 7.42, 7.37 and 6.37 respectively. The methyl angolensate has a methylene group (C=CH_2_) (CH_2_-30), the original bands at δ 5.13 and 4.88 being due to its protons. The presence of a double bond requires that methyl angolensate has two carbocyclic rings. The proton attached to C-1 carbon appeared as double doublet signal at δ 3.51 due to coupling with the neighboring hydrogen atoms at C-2 (Fig. 2). A survey of the literature revealed that the physical and NMR spectral data of crystalline needles of fraction A were in good agreement with those recorded for methyl angolensate (21-23). Methyl angolensate was identified by comparision of their mp, IR and ^1^H NMR data with reported values as well as by a detailed analysis of the ^1^H and ^13^C spectral data and the bond connectives were also clearly established. The infrared spectrum of methyl angolensate had a strong band at 1735 cm^-1^ indicating the presence of an ester and/or lactone function and showed the absence of a hydroxyl group. Bevan and his co-workers established the presence of methoxycarbonyl group. The currently accepted molecular formula, C_27_H_34_O_7_ was proposed by Housley (24). Hence we conclude that compound isolated from fraction A is methyl angolensate (Fig 3A).

Methyl angolensate has been isolated from several species of the family Meliaceae (24-26) in which it is present in variable amounts together with gedunin (27), dihydrogedunin, and entandrophragmin and utilin (26) and has also been obtained from the seeds of *Swietenia mahagoni* (23), wood extractives of *Khaya grandifolia* and *K. ivorensis* and stem bark of *K. anthotheca* (28), from stem bark of *Entandrophragma angolense* (29), *Khaya grandifolia* (30), ethyl acetate seed extract of *Neobegueae mahafalensis* (31) and also from the seeds of *Carapa guianensis* (32).

The yellow amorphous powder obtained from fraction B was analyzed by ^13^C NMR spectrum, which showed signals for 21 carbon atoms. The IR spectrum also showed two strong absorption bands at 3365 and 1652 cm^-1^ indicating the presence of hydroxyl and conjugated carbonyl groups, respectively.

IR (KBr) ν_max_: 3365 (-OH), 1652 (C=O), 1591 (C=C), 1565, 1455 cm^-l^.

**Figure 4:**
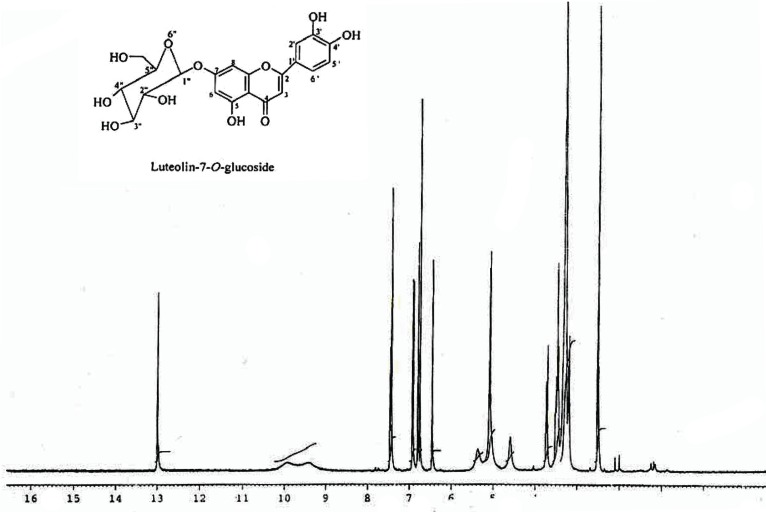
^1^H NMR sprctra of luteolin-7-*O*-glucoside (400 MHz, DMSO-d6) showing a series of signals between δ 3.73-3.15, attributable to a sugar moity and the down field signal at δ 12.98 (1H, s) and two meta-coupled doublets (*J* = 1.9 Hz) at δ 6.79 and 6.45, each integrating for one proton, assigned to H-8 and H-6. The presence of ABX system at δ 7.46 (dd, *J* = 8.3, 2.0 Hz), 7.43 (d, *J* = 2.0 Hz) and 6.92 (d, *J* = 8.3 Hz), characteristic of 1, 2, 4-trisubstituted phenyl unit. Only singlet at δ 6.75, integrating for one proton, was attributed C-3 to proton of flavonoids.

^1^H NMR (Fig. 4): d 12.98 (1H, s, OH-5), 7.46 (1H, dd, *J* = 8.3, 2.0 Hz, H-6′), 7.43 (1H,d, *J* = 2.0 Hz, H-2′), 6.92 (1H, d, *J* = 8.3 Hz, H-5′), 6.79 (1H, d, *J* = 1.9 Hz, H-8), 6.75 (1H, s, H-3), 6.45 (1H, d, *J* = 1.9 Hz, H-6), 5.09 (1H, d, *J* = 7.3 Hz, H-1′′), 3.73-3.17 (5 H, m, H-2″,3″, 4″, 5″ and 6″).

**Figure 5:**
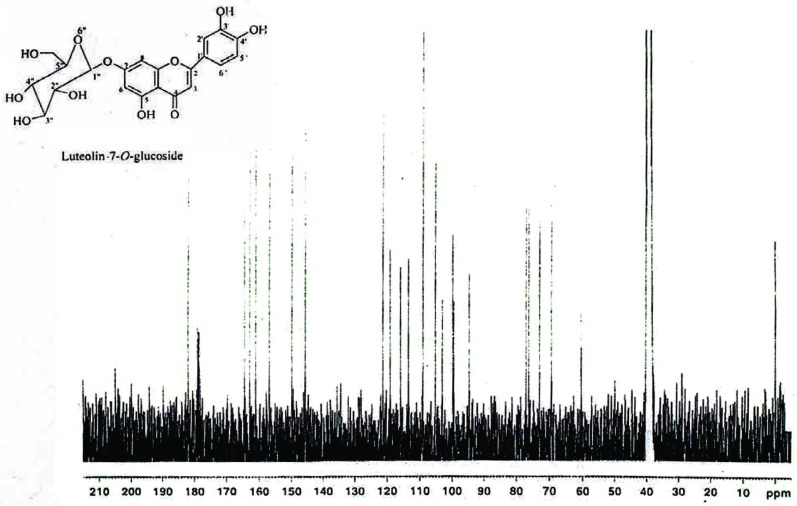
^13^C NMR spectra of luteolin-7-*O*-glucoside (75 MHz, DMSO-46) data showing the presence of a ketone carbonyl (δ 181.8), two olefinic carbons (δ 164.3 and 103.0), four gydroxyl carbons (δ 162.8, 161.0, 149.8 and 145.7). The coupling constant (*J* = 7.3 Hz) of the anomeric proton located at δ 5.09 and the ^13^C NMR chemical shifts of the sugar carbons (δ 99.7, 77.0, 76.3, 73.0, 69.4 and 60.5) revealed the presence of β-*O*-glucoside unit.

^13^C NMR (75 MHz, DMSO- *d*_6_) (Fig. 5): δ 181.8 (C-4), 164.3 (C-2), 162.8 (C-7), 161.0 (C-5), 156.8 (C-9), 149.8 (C-4′), 145.7 (C-3′), 121.2 (C-1′), 119.0 (C-6′), 115.8 (C-5′), 113.4 (C-2′), 105.2 (C-10), 103.0 (C-3), 99.7 (C-1″), 99.4 (C-6), 94.6 (C-8), 77.0 (C-5″), 76.3 (C-3″), 73.0 (C-2″), 69.4 (C-4″), 60.5 (C-6″).

The ^1^H NMR spectrum of yellow amorphous powder showed a down field signal at δ 12.98 (1H, s) indicating the presence of a chelated hydroxyl at C-5 position. It also showed two meta–coupled doublets (*J*=1.9 Hz) at δ 6.79 and 6.45, each integrating for one proton, and were assigned to H-8 and H-6, respectively of ring A of 5,7-dihydroxyflavonoids (33). The presence of ABX system at δ 7.46 (dd, *J* = 8.3, 2.0 Hz), 7.43 (d, *J* = 2.0 Hz) and 6.92 (d, *J* = 8.3 Hz), characteristic of 1, 2, 4-trisubstituted phenyl unit (33). The only singlet at δ 6.75, integrating for one proton, was attributed C-3 to proton of flavonoids (35). These spectral data revealed the presence of luteolin skeleton (36). In addition, the ^1^H NMR spectrum showed a series of signals between δ 3.73-3.15, attributable to a sugar moiety (Fig. 4). The coupling constant (*J* = 7.3 Hz) of the anomeric proton located at δ 5.09 and the ^13^C NMR (Fig. 5) chemical shifts of the sugar carbons (36) (δ 99.7, 77.0, 76.3, 73.0, 69.4 and 60.5) revealed the presence of β-*O*-glucoside unit in luteolin-7-*O*-glucoside. The ^13^C NMR data showed the presence of a ketone carbonyl (δ 181.8), two olefinic carbons (δ 164.3 and 103.0), and four hydroxyl carbons (δ 162.8, 161.0, 149.8 and 145.7). On comparison with the literature on flavonoids of luteolin, it was revealed that the physical and spectral data of luteolin-7-*O*-glucoside isolated earlier from *Vitex agnus castus* (38) and *Vernonia cinerea* (19) were in good agreement with those recorded for luteolin-7-*O*-glucoside (Fig. 3B) in the present study (37). Therefore, we conclude that compound isolated from fraction B is luteolin-7-*O*-glucoside.

Even though methyl angolensate (Fig. 3A) and luteolin-7-*O*-glucoside (Fig. 3B) were reported from the other sources (19, 20), this is the first report for the occurrence of these compounds from the callus of *S. febrifuga* and also family Meliaceae.

### Antimicrobial activity of purified compounds

**Figure 6:**
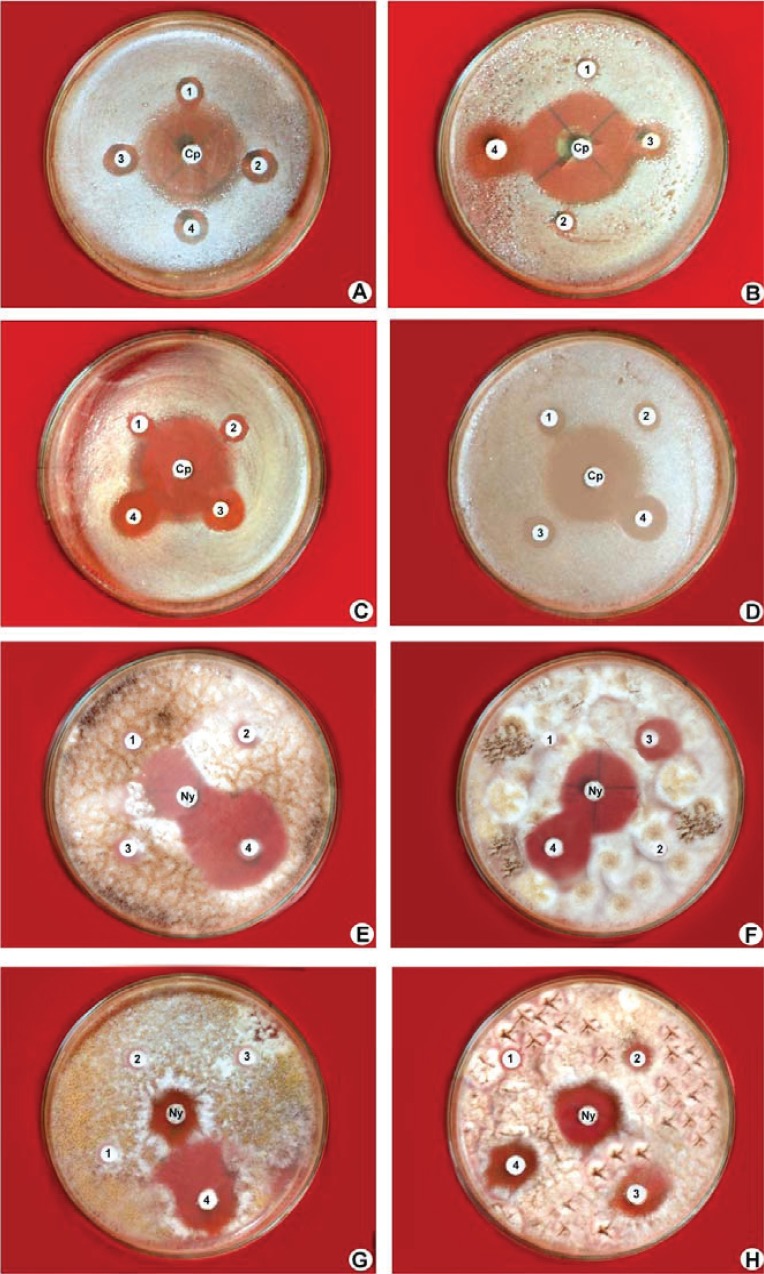
Antimicrobial activity of methyl angolensate and luteolin-7-*O*-glucoside by Disc diffusion assay. **A & B,** Antibacterial activity of methyl angolensate against *Proteus vulgaris* and *Bacillus subtilis* at various concentrations (1) 50 μg/disc (2) 100 μg/disc (3) 200 μg/disc (4) 400 μg/disc and cp-ciprofloxacin (centre); **C & D,** Antibacterial activity of luteolin-7-*O*-glucoside against *Proteus vulgaris* and *Salmonella typhimurium* at various concentrations (1) 50 μg/disc (2) 100 μg/disc (3) 200 μg/disc (4) 400 μg/disc and cp-ciprofloxacin (centre); **E,** Antifungal activity of methyl angolensate at various concentration (1) 50 μg/disc (2) 100 μg/disc (3) 200 μg/disc (4) 400 μg/disc against *Aspergillus niger* showing zone of inhibition more than the standard, nystatin (centre); **F-H,** Antifungal activity of luteolin-7-*O*-glucoside at various concentrations (1) 50 μg/disc (2) 100 μg/disc (3) 200 μg/disc (4) 400 μg/disc against *Alternaria alternata, Aspergillus fumigatus, Aspergillus niger* along with the standard, nystatin (centre).

The *in vitro* antimicrobial activity of methyl angolensate and luteolin-7-*O*-glucoside against microorganisms and their activity potentials were qualitatively assessed by disc diffusion assay. In this assay, the presence or absence of inhibition zones and zone diameters were analysed. The results of the antibacterial activity and antifungal activity are presented in Table 3. Methyl angolensate showed considerable antibacterial activity at both the concentrations of 200 and 400 μg/disc compared to luteolin-7-*O*-glucoside. However luteolin-7-*O*-glucoside promoted considerable antifungal activity at concentrations of 200 and 400 μg/disc compared to methyl angolensate (Table 3, Fig. 6).

**Table 3:** Antimicrobial activity of methyl angolensate and luteolin-7-*O*-glucoside

Name of the organism	Diameter of the zone of inhibition (mm)				
	Standard	Methyl angolensate		Luteolin-7-*O*-glucoside	
		I	II	I	II

*Klebsiella pneumoniae*	31.2 ± 0.67	10.4 ± 0.43	13.5 ± 0.41	10.0 ± 0.16	12.5 ± 0.67
*Salmonella typhimurium*	26.1 ± 0.53	9.10 ± 0.25	12.0 ± 0.64	10.5 ± 0.50	14.3 ± 0.00
*Proteus vulgaris*	22.8 ± 0.27	10.5 ± 0.88	14.1 ± 0.12	11.1 ± 0.36	13.0 ± 0.25
*Pseudomonas aeruginosa*	17.5 ± 0.38	9.30 ± 0.10	12.5 ± 0.78	-	11.3 ± 0.37
*Bacillus subtilis*	28.0 ± 0.18	11.5 ± 0.18	15.2 ± 0.50	10.4 ± 0.70	13.5 ± 0.14
*Escherichia coli*	31.4 ± 0.22	10.0 ± 0.52	12.8 ± 0.33	11.6 ± 0.34	11.0 ± 0.43
*Staphylococcus aureus*	34.2 ± 0.10	10.2 ± 0.73	13.3 ± 0.67	-	10.4 ± 0.52
*Aspergillus fumigatus*	14.4 ± 0.20	-	11.4 ± 0.25	10.2 ± 0.15	17.5 ± 0.42
*Aspergillus niger*	17.2 ± 0.13	10.6 ± 0.15	17.3 ± 0.49	11.7 ± 0.38	14.6 ± 0.13
*Alternaria alternata*	23.1 ± 0.38	-	10.2 ± 0.19	15.2 ± 0.83	18.2 ± 0.67

Values are the mean of triplicates ± SE. Zone of inhibition including the diameter of the disc (6.0 mm). I, 200 μg/disc; II, 400 μg/disc. -, No inhibition; Standard, Ciprofloxacin (10 g/ml) for bacteria; Nystatin (500 g/ml) for fungi. Methyl angolensate (10 mg/ml); Luteolin-7-*O*-glucoside (10 mg/ml).

Results further demonstrated that methyl angolensate had the greatest effect on the growth of *Bacillus* (Fig. 6B) (15.2) followed by *Proteus* (14.1) (Fig. 6A), *Klebsiella* (13.5), *Staphylococcus* (13.3), *E. coli* (12.8) and *Salmonella* (12.0) at higher concentrations (Table 3). The luteolin-7-*O*-glucoside showed considerable growth inhibition of *Salmonella* (Fig. 6D) (14.3) when compared to methyl angolensate. Methyl angolensate has displayed the highest antifungal activity against *Botrytis cinerea* Pers (39) and is antimalarial (30), anti-inflammatory (40), antiallergic (32), used as antiulcer agent (29). Methyl angolensate also possess insect antifeedant properties (41) and spasmolytic activity in mice (42). Methyl angolensate has been shown to reduce spontaneous motor activity and exploratory activity in mice. This suggests that methyl angolensate is one of the biologically active principles having sedative effect (43). In the present study, luteolin-7-*O*-glucoside revealed significant inhibition of gram negative bacteria (*Salmonella, Proteus*) (Fig. 6C, D) with the exception of *P. aeruginosa* which are in agreement with Sousa *et al.* (44); Pereira *et al.* (45). However, low concentrations of luteolin-7-*O*-glucoside in the present study showed no activity against *Staphylococcus* and *Pseudomonas* but had activity against remaining tested bacteria. Ciprofloxacin, a broad-spectrum antibacterial drug for which most gram-negative bacteria are highly susceptible and many gram-positive bacteria are moderately susceptible. Most of the bacteria in the present study are gram negative; hence ciprofloxacin was selected instead of polymixin. The antibacterial activity of terpenoid and flavonoid is probably due to the membrane disruption by the terpenes (46, 47) and their quealting ability of flavones to form complex with extracellular and soluble proteins and with bacterial cellwalls hence disrupting the microbial membranes (47).

Among the fungal species tested, methyl angolensate displayed the maximum zone of inhibition (17.3) against *Aspergillus niger* (Fig. 6E) while the luteolin-7-*O*-glucoside exhibited maximum zone of inhibition (32.0) on *Alternaria alternata* (Fig. 6F). Luteolin-7-*O*-glucoside possesses antioxidant activity (48, 49) and at low concentrations suppresses the production of nitric oxide and prostaglandin in bacterial lipopolysacharide activated mouse macrophage cells without introducing cytotoxicity (50). The current study revealed that the inhibitory zones produced by methyl angolensate and luteolin-7-*O*-glucoside against bacteria was considerably lesser than that of the standard, ciprofloxacin, a broad-spectrum antibacterial drug to which most gram-negative bacteria are highly susceptible and many gram-positive bacteria are susceptible or moderately susceptible (51). The inhibitory zones produced by methyl angolensate and luteolin-7-*O*-glucoside against *Aspergillus niger and A. fumigatus* was considerably larger than or similar to that of the standard nystatin, the most commonly used standard antifungal agent (52).

Methyl angolensate and luteolin-7-*O*-glucoside have gained economic importance due to their anti-malarial, anti-inflammatory and anti-oxidant properties. In the present study, we have established conditions for production of these compounds from root callus of *S. febrifuga*. This is the first study to provide data that methyl angolensate and luteolin-7-*O*-glucoside possess potential antibacterial and antifungal activities as seen from its activity against wide range of microorganisms. Further research is necessary to establish the pharmacological mechanisms of each compound.
